# Understanding HIV-Related Mental Health Challenges and Contributing Factors Among Indonesian Adolescents Living with HIV

**DOI:** 10.3390/ijerph22010083

**Published:** 2025-01-09

**Authors:** Paul Russell Ward, Reni Puspitasari, Aasha Rose, Biniyam Sahiledengle Gebremariyam, Nelsensius Klau Fauk

**Affiliations:** 1 Centre for Public Health, Equity and Human Flourishing, Torrens University Australia, Adelaide 5000, Australia; paul.ward@torrens.edu.au (P.R.W.); biniyam.gebremariyam@student.torrens.edu.au (B.S.G.); 2 Sekolah Tinggi Ilmu Kesehatan Bethesda Yakkum, Yogyakarta 55224, Indonesia; reni@stikesbethesda.ac.id; 3 School of Education, University of Southern Queensland, Toowoomba 4350, Australia; aasha.rose@unisq.edu.au

**Keywords:** mental health, contributing factors, stigma, discrimination, ALHIV, qualitative, Indonesia

## Abstract

Human Immunodeficiency Virus (HIV) has disproportionately affected various population groups, including adolescents living with HIV (ALHIV). In many contexts, ALHIV have been reported to experience mental health issues following their HIV diagnosis. However, there is a limited understanding of the mental health issues faced by ALHIV in Indonesia and the various contributing factors globally. This study aimed to explore the mental health challenges and their contributing factors among Indonesian ALHIV. A qualitative design employing one-on-one in-depth interviews was used to collect data from ALHIV (*n* = 20) in Yogyakarta, Indonesia. Participants were recruited using the snowball sampling technique, beginning with the dissemination of study information sheets through a healthcare facility that provides HIV care services and via a WhatsApp group for adolescents living with HIV. The data were thematically analyzed, guided by a qualitative data analysis framework. The findings showed that ALHIV experienced a variety of mental health challenges upon learning of their HIV-positive status. Their mental health was also influenced by a range of family-related factors, stigma, and discrimination, which were also facilitated by their specific situations and settings, including living in a shared house with parents and siblings and school setting where they met and interacted with different peer groups on a daily basis. Family-related factors, including broken homes, family conflicts, lack of family support, and being orphans, negatively impacted their mental health. The awareness of perceived and anticipated stigma, and the experience of enacted stigma or discrimination, also contributed to the mental health challenges they faced. The findings indicate a pressing need for tailored and targeted HIV intervention programs and activities that support their mental health, reduce stigma, and promote HIV status disclosure in safe ways for ALHIV both within the study setting and beyond.

## 1. Introduction

According to the World Health Organization (WHO), adolescents are individuals in the age group of 10–19 years [1]. They are one of the population groups that have been disproportionately affected by the Human Immunodeficiency Virus (HIV) [2,3]. Of an estimated 39.9 million people living with HIV (PLHIV) globally reported in 2023, approximately 1 million were adolescents aged 15–19 years, with 140,000 newly diagnosed in 2023 [4,5]. The same report also estimates 14,000 AIDS-related deaths among adolescents in this age group in 2023 [4,5]. In Asia and the Pacific region, where Indonesia is located, around a quarter of the 300,000 new HIV infections in 2023 were diagnosed among adolescents and young people aged 15–24 years [6]. In Indonesia, the national AIDS report shows a total of 566,707 PLHIV, of whom 57,299 were newly diagnosed in 2023. Among these, 5.5 per cent are adolescents aged 15–19 years, which increased from 3.9 per cent in 2022 [7]. Of the 2023 newly diagnosed cases, 45 per cent were transmitted through sexual contacts, 43 per cent were unknown means of transmission, and 12 per cent were spread through other means of transmission, including mother-to-child transmission and injecting drug use [7].

Studies in different settings globally have reported that Adolescents Living with HIV (ALHIV) experience an increased burden of mental health challenges (e.g., depression, anxiety, attention-deficit hyperactivity disorder, post-traumatic stress disorder (PTSD), etc.) upon learning of their HIV status, which affect their thoughts, feelings, emotions, behaviors, and interactions with others [8,9,10,11,12]. For example, a 2021 systematic review and meta-analysis involving 10 studies from 8 countries reported a depression prevalence of 26.07% among ALHIV [13]. Other studies in developed countries, including France, Italy, Poland, Canada, and the US, found that depression, anxiety, attention-deficit hyperactivity disorder, and PTSD were more prevalent among ALHIV compared to their HIV-negative peers [8,14,15]. Similarly, in low- and middle-income countries (LMICs), accumulating evidence suggests that ALHIV face various mental health challenges after their HIV diagnosis [9,16,17]. For instance, studies in Africa, where an estimated 80% of ALHIV reside, reported that anxiety disorders, depression, fear, and PTSD are common among this population [9,10,16,18,19]. Additionally, suicidal ideation and attempts are also identified as mental health issues among these adolescents in several countries, including Kenya, Uganda, and South Africa [11,19,20,21].

The WHO has suggested that in addition to the HIV diagnosis itself, biomedical, familial, economic, and social or environmental factors encountered by ALHIV within their families and communities may increase the risk of mental health issues [22]. Limited evidence from studies in Malawi and South Africa shows that HIV stigma and discrimination, reflected in rejection and physical and emotional abuse within family, community, school, and healthcare settings, are associated with mental health issues in ALHIV [19,23]. HIV stigma and discrimination may not only increase the risk of mental health challenges among ALHIV but also serve as significant barriers to accessing and adhering to HIV treatment or antiretroviral therapy (ART), potentially resulting in unsuppressed or increased viral load and poor health outcomes [24,25]. Low household income and caregiver mental health problems are also reported to be contributing factors for depressive symptoms among ALHIV in Thailand and Cambodia [26]. Similarly, having both parents pass away is associated with increased anxiety symptoms among ALHIV in Indonesia [27]. Additionally, a recent study in Nigeria reported that alcohol consumption is associated with higher mental health scores among ALHIV compared to those who do not consume alcohol [28].

Despite the reported mental health challenges among ALHIV, there is a lack of evidence in the literature regarding the specific factors that contribute to these challenges. Moreover, most studies exploring HIV-related mental health issues in ALHIV in LMICs have primarily been conducted in African countries [11,16,29], with only a few being carried out in Malaysia, Thailand, Vietnam, Cambodia [26,30,31], China [32], and Indonesia [27]. This indicates that despite the increasing number of HIV infections among adolescents in Asia, there is a lack of evidence on mental health challenges and the contributing factors to ALHIV in the region [6]. In Indonesia, although there has been a more than 300-fold increase in HIV infections over the last decade [6,7], evidence on HIV-related mental health challenges and their contributing factors among ALHIV is lacking [27,33,34]. Within Indonesian society, HIV and PLHIV are highly stigmatized, as evidenced by negative social perceptions and moral judgments. HIV is often viewed as an infection associated with deviant behaviors and regarded as a curse, while PLHIV are seen as individuals with low moral standards, “trash people”, and sinners [35,36]. Such perceptions and judgments appear to be rooted in, or supported by, certain religious values and beliefs that regard extra- or pre-marital sex and sexual relations with multiple partners as sinful, and HIV as a curse for PLHIV as it is intertwined with human sexuality [35,37]. Moreover, the communal characteristics and strong community ties within Indonesian society appear to facilitate the spread of these negative social perceptions and moral judgments across various settings, including families, communities, schools, workplaces, and healthcare settings [38,39]. Therefore, for ALHIV in Indonesia, their frequent social interactions with peers at school and within their communities, and who still live with parents and siblings in shared houses, these contexts can significantly shape their experiences of living with HIV, as well as their emotions, mental health, and social relationships, in ways that differ from those of adults living with HIV. This study aimed to address this gap by exploring in-depth the views and experiences of ALHIV in Yogyakarta, Indonesia, regarding the mental health challenges they faced upon learning of their HIV status and the contributing factors. Understanding the views and real-life experiences of ALHIV can be useful to inform policies and the development of tailored approaches, intervention programs, and various activities that support their mental health, reduce stigma, and promote HIV disclosure in safe ways for ALHIV in the study setting and similar contexts in Indonesia and beyond [40].

## 2. Materials and Methods

### 2.1. Study Design and Participant Recruitment

A qualitative phenomenological study design [41] employing in-depth interviews was used to explore participants’ views and experiences of HIV-related mental health challenges and factors that contributed to the challenges. The use of this qualitative design was useful since it enabled the researchers to probe deeply into how respondents viewed their experiences of HIV-related mental health challenges within situations and settings (family, school, community, healthcare settings) where they lived and interacted with other people, and how they understood factors that contributed to those challenges. The qualitative design has been described to be effective in studying participants’ views in their settings and can lead to a deeper insight into their real-life experiences [42].

The study participants were ALHIV from urban communities in Yogyakarta municipality, Indonesia. This setting was selected because of feasibility, familiarity, and the potential of undertaking the current study successfully. They were recruited using a combination of the purposive (to recruit initial participants) and snowball sampling techniques. The snowball technique was employed due to the challenges associated with accessing this hard-to-reach population (ALHIV). This approach was necessitated by the sensitivity of the topic under investigation, the high vulnerability of this group, and the widespread stigma surrounding HIV that affects PLHIV in the study setting [43,44]. The study information sheets, which also contained the contact details of the field researcher (RP), were initially distributed to potential participants and/or their parents/guardians through the receptionist and information board at a healthcare facility providing HIV care services in the study setting and via a WhatsApp group of adolescents living with HIV. The field researcher approached the head of a healthcare facility providing HIV care services in the study setting and the coordinator (a companion of PLHIV who is also living with HIV) of a WhatsApp group for initial conversations regarding the possibility of the distribution of study information sheets to potential participants. Some initial participants who contacted the field researcher and confirmed their participation in the study were recruited for an interview. This was then followed by the snowball technique where, at the end of the interviews, they were asked to provide multiple other referrals and help distribute copies of the study information sheets to their eligible friends who might be willing to participate in this study [43]. Participants were recruited based on several inclusion criteria. They had to be: (i) an adolescent living with HIV, (ii) aged between 16 and 18 years, and (iii) willing to voluntarily participate in this study. For participants under 18 years, consent from their parents or guardians was sought before their participation. The study information sheets and consent form were sent via WhatsApp, email, or provided in person by the field researcher. When the participant was under 18, both they and their parents/guardians signed written consent forms.

### 2.2. Data Collection

Data collection was conducted with ALHIV using in-depth interviews from June to October 2023. They were interviewed online via WhatsApp or phone calls, while some were interviewed offline in person in a private room at their homes. The options for both offline and online interviews were made available to minimize barriers to participation and to allow the participants to choose the mode of interview that best suited them. In addition, most online interviews were carried out via WhatsApp video calls, which enabled the researcher to observe non-verbal language throughout conversations with the participants. Time, venue, and modes of interview were agreed upon by the field researcher or interviewer (RP/female) and each participant during initial contact for participation confirmation. The field researcher is a lecturer and researcher who has undergone formal training in qualitative research methods during her master’s program (M.Kes). She has research experience in various topics related to public health and nursing topics, including HIV. Interviews were conducted in Bahasa Indonesia (Indonesian) and audio recorded. Interviews were focused on several main areas, including the participants’ account of how they contracted HIV and how they were informed or knew about their HIV status, their views and experiences of mental health challenges upon learning of their HIV status, their lived experiences at home, in their community, at school, or on campus after the diagnosis, and their views on factors that also contributed to mental health challenges they faced. The interview guide was developed in English and then translated into Indonesian (Supplementary File S1: Interview Guide). Both versions went through several revisions based on comments from the researchers and from the Human Research Ethics Committee, Torrens University Australia in Australia and Medical and Health Research Ethics Committee, Krida Wacana Christian University in Indonesia. Interviews took approximately 35–50 min. Two participants were accompanied by their mothers at their request during the interviews. The number of participants or interviews was determined based on whether the collected data or information had been rich enough to address the research aim and whether data saturation had been reached. Data saturation was indicated in the similarity of information provided by the last few participants, suggesting that data collection had reached the point where no new insights were emerging, indicating that an adequate sample size had been reached [45]. Finally, twenty adolescents were recruited and interviewed in this study. Before participating in the interviews, the consent forms were returned to the field researcher, indicating the participants’ voluntary participation. Some returned the form in person on the interview day while others sent it back to the researcher through email or WhatsApp. No repeated interviews were conducted with any of the participants. Participants and researchers had no established relationships prior to the study. The interview transcripts were not returned to participants to seek their corrections and comments given the sensitive nature of this research topic and to avoid the possibility of the transcripts of participants who had not been open about their HIV status being received and read by their family members.

### 2.3. Data Analysis

Before commencing the comprehensive analysis of the data, the audio recordings of the interviews were transcribed verbatim. Data analysis had been started alongside the data collection process. Transcriptions were made following each interview, which facilitated the direct integration of field notes into each transcript (RP and NKF). This also enabled the researchers to appreciate the richness of the information or data provided by the participants and to determine whether data saturation had been reached. Although the data analysis was carried out by NKF, ongoing discussions and sharing of the progress were maintained with other researchers throughout the analysis and manuscript drafting phases, during which revisions and restructuring of the themes occurred. Data analysis was conducted in Indonesian to preserve the social meaning attached to the participants’ narratives. For the purpose of this publication, selected quotes were translated into English by NKF, who is fluent in Indonesian and English, and then reviewed by RP who is also fluent in both English and Indonesian. To ensure accuracy, comparisons of the original Indonesian transcripts with the English translations were consistently performed throughout the data analysis and manuscript writing stages [46]. Additionally, other authors (PRW, AR, BSG) reviewed the English translations during the manuscript drafting and revision processes to maintain quality. The analysis followed a qualitative data analysis framework [47], offering a systematic approach to data management enhancing the rigor, transparency, and validity of the analytic process.

The analysis was performed by repeatedly listening to each audio recording and reading the transcript. At this stage, the narrative in each transcript was segmented into smaller data extracts and labels, notes, and comments were added. In the following step, key concepts and issues identified from the transcripts were listed to create a thematic framework. This process was iterative, involving continuous refinement of the themes based on whether there were sufficient coded data extracts or information to support them. Next, data indexing was performed by assigning open codes to data extracts. This was followed by close coding, involving identifying and grouping similar or redundant codes into overarching themes and sub-themes. For example, codes assigned to participants’ accounts regarding self-isolation, feelings of anger and sadness, denial of their HIV status, overthinking, worry, depression, and suicidal ideation were collated under the theme “HIV-related mental health challenges facing ALHIV”. Codes reflecting familial factors, such as broken homes, family conflicts, lack of family support, and being orphans, that negatively impacted the mental health condition of ALHIV were grouped under the theme “Individual factors contributing to mental health challenges faced by ALHIV”. Codes on perceived stigma, anticipated stigma, and enacted stigma faced by these adolescents within their family, community, school, or healthcare settings that affected their mental health were grouped under the theme “Stigma, discrimination, and mental health challenges faced by ALHIV”. The last theme was initially named “Social factors and mental health challenges among ALHIV”, but in the end, it was revised to the current one to be precise. The findings (codes and themes) were consistently compared both within and across interviews during the data analysis and writing stages. Ultimately, all the data were mapped and interpreted, and the final agreed-upon themes and interpretations are presented in this manuscript.

### 2.4. Ethical Consideration

Ethics approvals for this study were obtained from Human Research Ethics Committee, Torrens University Australia, Australia (No. 0214, 01/12/2022) and Medical and Health Research Ethics Committee, Krida Wacana Christian University, Indonesia (No. SLKE: 1327/SLKE-IM/UKKW/FKIK/KE/VIII/2022). Before commencing interviews, participants again received a verbal explanation from the interviewer about the aim of the study and the future use of the data or information they provided. They were informed that their participation was voluntary and there would be no consequences if they withdrew their participation or information (data) before, during, or after the interview. They were also advised that the interviews would be audio-recorded, any personal information would be removed from the transcripts to maintain the anonymity and confidentiality of the information, and pseudonyms would be used. All the names attached to the direct quotes under the ‘Results’ section are pseudonyms. Given the topic of the study and the potential for emotional discomfort during the interviews, these adolescents were informed that an HIV counsellor, whom they had previously consulted, and support from a companion for individuals living with HIV were available at no cost should they require assistance.

## 3. Results

The adolescents interviewed in this study comprised 6 girls and 14 boys, aged between 16–18 years; 12 were 18 years old, 5 were 17, and 3 were 16 years old. Eight were still in high school, six graduated high school, and the others recently enrolled in university. Eight adolescents acquired HIV through mother-to-child transmission (MTCT), while twelve were infected through sexual contact, with five boys acquiring it through homosexual contact or sex with men. Most of the participants had been diagnosed with or known about their HIV status for several years, while six were newly diagnosed with the infection within a few months of the interviews.

The findings presented below are organized into three main themes: (i) HIV-related mental health challenges facing ALHIV, (ii) Family-related factors associated with health challenges in ALHIV, and (iii) Stigma, discrimination, and mental health challenges faced by ALHIV. The themes are explained in detail below.

### 3.1. HIV-Related Mental Health Challenges Facing ALHIV

The diagnosis of HIV and living with the condition significantly affected the mental health of the adolescents interviewed in this study. Both female and male participants shared their experiences with various HIV-related mental health challenges, including self-isolation, daydreaming, feelings of anger and sadness, denial of their HIV status, overthinking, anxiety, depression, and suicidal thoughts. Ayu, a 16-year-old girl, described how she often “isolated” herself and “daydreamed” in her room after discovering her HIV positive status, which she viewed as something negative, despite not fully comprehending it at the time. A similar experience was recounted by Pram, an 18-year-old boy who contracted the virus through heterosexual contact. Following his HIV diagnosis, he “isolated” himself in his room due to confusion about his situation and a lack of understanding regarding the actions he could take. Retreating to his room became a means for him to avoid interacting with others, including his own family members, and to escape inquiries about his HIV status.

*“I often isolated myself in my room, daydreamed, and my mind was blank because I couldn’t fully understand what I was experiencing even though I knew that this was something negative”*.

(Ayu)

*“I was very confused at that time (early stage of HIV diagnosis), not knowing what I should do. I also didn’t want to meet other people, didn’t want to talk to anyone, fearing that they would ask about my condition. That’s why I just locked myself away”*.

(Pram)

Furthermore, Aditya, who contracted HIV through heterosexual relations, tended to “overthink” his positive HIV status and felt “worried” and “afraid” after being diagnosed with HIV, as his HIV status constantly lingered in his mind. Not only that, but he was also perpetually “worried” about the possibility of others discovering his HIV status and endeavored to reassure himself by asking healthcare professionals about the confidentiality of his status. The experience he went through highlights the burden of mental health challenges, especially during the initial stage of HIV diagnosis:

*“I try to just go through it, but I still often think about why my situation is like this. I am afraid to be open (with others) about my condition. Every day I feel worried and wonder what if others don’t understand my situation”*.

(Aditya)

The self-isolation by Ayu, Pram, and others appeared to also be influenced by the circumstances of living with their parents and siblings or extended family members in shared house, which complicated their ability to avoid interactions and conversations with others in the family and to be free from parental supervision. Such circumstances not only placed intense pressure on them but also led to increased difficulties in concealing their HIV status from their parents or siblings, as experienced by Pram, Aditya, and others such as Richard, who was diagnosed two months prior to the interview, and Silvia, who lived with her extended family after her parents passed away. These circumstances created difficulties for them to feel free in managing their health conditions, engaging in activities of their choosing, and making decisions to support their mental health as adults living with HIV would do:

*“I hide my medication from them all because I do not want them to know (about my HIV status). I conceal the medicines in the seams of my clothes in the wardrobe. Sometimes I feel so worried if they (his family members) come into my room …”*.

(Richard)

*“I carefully keep it (his/her HIV status) secret so that they (her extended family) do not find out…”*.

(Silvia)

Wisnu, a 17-year-old boy who contracted HIV through homosexual contact with other men, also faced significant mental health challenges. He experienced prolonged periods of depression, during which suicidal thoughts frequently occupied his mind. He contemplated “making it fast” (suicide) by jumping from a bridge or throwing himself in front of a train. His depression and suicidal ideation seemed to be exacerbated by his awareness of the prevalent social perceptions of HIV as a “taboo”, “disgusting”, and “scary” infection, which he appeared to internalize. Furthermore, the fact that he contracted HIV through same-sex contact seemed to add another layer of burden, making it difficult for him to be open about his situation with his family. Wisnu’s story highlighted the unacceptance of HIV, PLHIV, and homosexuality within communities and reflected the heightened difficulties of living with HIV:

*“The issue of HIV in Indonesia is truly taboo. It is very saddening when someone is infected with HIV, as many people feel fear and disgust towards the condition. At that time, I was indeed experiencing a prolonged period of depression. According to the doctor, I was suffering from moderate depression. I even contemplated suicide, considering jumping from a high bridge or throwing myself in front of a train to end my life instantly. I just wanted all of this to be over quickly”*.

(Wisnu)

Wisnu’s and others’ awareness of the various negative social perceptions surrounding HIV, PLHIV, and homosexuality was further compounded by their specific situations as adolescents, where they had to attend school, meeting and interacting with different peer groups on a daily basis. Yeni, a high school student, clearly described such a situation that left her constantly worried and afraid of the possibility of her schoolmates finding out about her health condition. This circumstance has made her very selective in choosing friends at school to avoid such a possibility. A similar experience was shared by Heri, who admitted to feeling uncomfortable and overthinking the questions posed by some of his classmates regarding his health every time he requested leave for medical reasons:

*“I am now in senior high school and every day I go to school, meet, and mingle with my friends at school, but I often feel worried and afraid that some might find out about my health condition. …. I know that many people are scared of HIV, which is why I have been cautious in choosing friends at school”*.

(Yeni)

*“I go to the hospital to collect my medication (ART) every month and usually inform the school that I am ill. My friends often ask, “What illness do you have?” This makes me feel uncomfortable, and at times I find myself overthinking it. I always try to come up with alternative reasons so that they do not know what I am experiencing”*.

(Heri)

### 3.2. Family-Related Factors Associated with Mental Health Challenges in ALHIV

The adolescents in this research recounted various family-related factors that contributed to the burden of mental health challenges they experienced. These included living in a disrupted family home, conflicts or disputes among family members, a lack of support from family members or parents, and the loss of both parents. These issues appeared to create extra difficulties for them as they were still living with their parents and were dependent on their support in many aspects, a situation which seems to be different to that of most adults living with HIV, who can manage their own lives independently. Ayu described her disrupted family home by referring to her parents’ divorce, a situation leading to her feeling of anger and struggle to accept her condition of living with HIV. A similar experience was shared by Gunawan, who was diagnosed with HIV three months prior to his interview. His parents were divorced and did not communicate with each other, a situation that made him feel alone and which he admitted increased the burden of the mental health challenges he faced:

*“My father transmitted HIV to my mother, then left us, neglecting us. I cannot accept this. We have to bear the consequences even though it is not my mother’s fault or mine. I am angry and frustrated”*.

(Ayu)

*“I come from a broken home; I feel alone carrying all this burden…”*.

(Gunawan)

Gunawan further described the characteristics of his family members. He realized that both his parents and his siblings were “temperamental”, which made him feel afraid and reluctant to be open about his HIV status with them. Similarly, Richard perceived the conflicts among his siblings within his family as a condition that heightened his fear and added to the burden of living with HIV. Such family situations appeared to leave these adolescents with no option but to face it, as they still lived with their parents and siblings and were unable to manage their lives independently. This circumstance further compounded the mental health challenges associated with managing HIV:

*“My siblings and I do not get along with each other. We are currently facing family problems, which is why I feel afraid and heavily burdened by my condition”*.

(Richard)

The lack of support from family members, particularly parents, was also a contributing factor to the mental health burden experienced by these adolescents. This was not only illustrated by Gunawan, Richard, and seven other adolescents who did not disclose their HIV status to their families, but also by others whose HIV status was known to their family members. For Joko, his family was aware of his HIV status; however, his mother and older brother did not accept his condition and did not provide the support he hoped for. This has left him feeling “very disappointed”. Pram had a similar experience, as he was ignored by both of his parents without any support for months after he informed them of his HIV status, a situation that left him feeling down and desperate. Considering that these adolescents lived with their parents and were not yet able to support themselves, their lived experiences highlight the significant importance of family support for ALHIV to cope with the HIV-related challenges they encountered and to navigate living with HIV:

*“My mother doesn’t accept (his HIV status) until now… My parents do not provide any support at all. This has made me feel very disappointed…”*.

(Joko)

*“I was ignored by my parents for several months, with no support whatsoever. I felt down and desperate at that time…”*.

(Pram)

Furthermore, homophobia within families by parents was another family-related factor that created difficulties for five adolescents who are men who have with men (MSM) in managing the mental health challenges facing them while at the same time concealing their homosexual orientation from their parents and others. Bambang, who contracted HIV through same-sex relationships, expressed that his sexual orientation is a significant concern for him. He felt anxious and afraid to inform his parents about his HIV status, as it might lead to questions regarding how he acquired the infection and potentially expose his sexual orientation. He further elaborated that same-sex relationships are heavily challenged within the community he comes from, including within his own family. The same fear was echoed by Chayo, a first-year university student. He believed that if his HIV status were to be discovered by his family, his parents would undoubtedly seek to understand how he contracted HIV. He feared that if his sexual orientation were revealed, it would become a source of shame for his family. This caused him considerable worry and fear, leading him to choose to live in a rented room and conceal his HIV status from others, including his family. Their narratives highlight the pervasive lack of acceptance of both HIV and same-sex relationships within their families and communities, as well as a significant absence of open communication with their parents regarding HIV or their sexual orientations:

*“I am afraid because same-sex relationships are not accepted; they are considered abnormal and condemned by people. I do not want my parents to know. I am now anxious about how to tell my parents about my HIV status”*.

(Bambang)

*“If my family, especially my father and mother, find out (about my HIV status), they will want to know how I got infected. I am very anxious; if they discover that I was infected through a same-sex relationship, it would be a disgrace for the family…”*.

(Chayo)

### 3.3. Stigma, Discrimination, and Mental Health Challenges Faced by ALHIV

Stigma and discrimination related to HIV significantly contributed to the mental health challenges faced by adolescents in this study. They experienced perceived stigma, anticipated stigma, and enacted stigma within their family, community, school, or healthcare settings, which affected their mental health. The specific settings they were in, including living with parents or extended families in a shared house, school setting with massive numbers of students, and different peer groups with whom they interacted on a daily basis posed an increased worry, fear, and challenge of the possibility of their HIV status being revealed. For instance, Silvia (an orphan) described the perceived stigma that is reflected in her awareness and understanding of how non-infected individuals behave towards or think about people living with HIV. She was aware of the potential “negative thoughts” from her extended family if her HIV status were to be revealed, leading her to feel “worried” and “afraid” and to conceal her HIV status from them. The fact that she did not feel close to her extended family members further reinforced her perceived stigma and her decision to hide her HIV status. Gibran, a 17-year-old boy who contracted the virus through unprotected heterosexual relations, was also aware that many people in society “are not yet able to accept” those living with HIV and hold negative views towards them. This made him “afraid” of his HIV status being discovered by his friends at school:

*“I am afraid and concerned that they (her extended family from her mother’s side) will think negatively about me, which is why I am not yet ready to inform them about my HIV status. They might be afraid to get close to me or even ask me to leave. I am not very close to them as I have only recently moved here…”*.

(Silvia)

*“There are still people who cannot accept a friend who is living with HIV. They do not want to associate with him because they fear transmission. There are still many negative perceptions about those living with HIV, which is why I am scared and hide my status; I do not feel brave enough to share it with my friends”*.

(Gibran)

Aditya echoed the same perceived stigma and explained his awareness of common social perceptions about people living with HIV as being “disgusting” and “dirty” due to various negative thoughts regarding HIV and how individuals contracted it. This made him feel fearful and ashamed if others were to know about his HIV status:

*“Many people feel disgusted (by those with HIV), considering them dirty. That’s what sometimes makes me anxious, scared, and ashamed…”*

Furthermore, Richard described his anticipation about the negative actions his siblings might take against him if his HIV status were to be revealed (anticipated stigma). The situation of living in the same house as his parents and siblings seemed to also shape his worry about the high possibility of them finding out about his HIV status and using it as a reason to attack him. Similarly, Gunawan feared that he could be subjected to physical abuse by his father if it were known that he was infected with HIV through sexual relations. This fear led him to conceal his HIV status from his family. Such experiences may not be encountered by adults living with HIV who are not in situations similar to those of these adolescents or those who do not live in shared housing with parents or extended family members:

*“I am afraid that this (his HIV status) will be used as a reason to attack me because we are having family problems. This is also one of the things that makes me worried too much”*.

(Richard)

*“My father could do things I don’t want, which is why I am scared and always on edge. I could be beaten if they (the family) find out that I have HIV and that I contracted it through sexual relations”*.

(Gunawan)

Enacted stigma, which is reflected in various discriminatory attitudes and behaviors exhibited by others, was also experienced by most of the participants. These negative attitudes often originated from parents, friends, teachers, and healthcare professionals, significantly impacting their mental health. Joko recounted how his mother would separate his food and eating utensils from those of others, which made him “feel sad”. While he disagreed with such treatment, Joko admitted that he felt powerless to do anything other than accept it. A similar narrative of HIV-related discrimination was shared by Ayu, who experienced being “avoided” and “bullied” by her friends at school due to HIV-related changes in her physical appearance or skin. Furthermore, she described other children who were not allowed by their parents to engage in certain activities or play with her in her community. Such discriminatory behaviors from others made her feel pressured and afraid to socialize. It is evident that the specific settings of the family house where they lived with parents and other family members and the school where they met and interacted with peers daily shaped and contributed to their experience of enacted stigma or discrimination against them and the mental health challenges they faced:

*“My mother always separates my food and eating and drinking utensils, and they are not allowed to be mixed with others. She always instructs others in the house to separate my eating utensils. I cannot accept this; I feel sad being treated this way, but I can only cry…”*.

(Joko)

*“When I was little, I was often avoided and bullied by my friends. My skin turned dark and there were wounds on my legs. They called me dirty. No one wanted to be near me. They refused to eat or play with me. Neighbors did not allow their children to visit me at my house. Sometimes I couldn’t sleep…. I felt pressured, and because of that, I didn’t like making friends…”*.

(Ayu)

Ganjar, a 16-year-old boy, described experiencing similar discrimination related to HIV at school from his teachers when they learned about his HIV status. This was reflected in the school’s decision to prevent him from attending in-person classes, instead instructing him to participate online. This situation led to mental health challenges, including feelings of isolation and stress due to his inability to interact with his peers. Discriminatory treatment was also experienced by Yanri, who recounted an incident where a healthcare professional loudly mentioned her HIV status in front of other patients at a healthcare facility, causing her embarrassment and concern that others would remember her status:

*“When the school principal and some teachers knew that I have HIV, they asked me to attend classes online. After a while, I began to feel lonely and unable to socialize with my friends; sometimes I felt stressed because I was suddenly cut off from them”*.

(Ganjar)

*“I felt extremely embarrassed when my HIV status was suddenly called out loudly. Everyone was looking at me”*.

(Yanri)

Yanri further shared her constant worry about the confidentiality of her HIV status every time she met or interacted with others, particularly in healthcare facilities, fearing that her HIV status might be known by those with whom she interacted.

## 4. Discussion

This study explored the mental health challenges faced by ALHIV, and various family-related factors, including stigma and discrimination, that contributed to those challenges. The findings highlight significant mental health challenges encountered by these individuals, including feelings of isolation, denial, sadness, anger, fear, worry, depression, and suicidal ideation upon learning of their HIV status. These are consistent with previous findings reporting prevalent mental health issues, such as depression, anxiety, PTSD, attention deficit hyperactivity disorder, and suicidal ideation and attempts among ALHIV [8,9,10,11]. Similar mental health challenges have also been reported among adults living with HIV in many different settings globally [48,49]. What the current study adds to the existing literature is the understanding of these adolescents’ specific settings and situations. These include living with their parents and siblings, or extended family members, in shared housing, attending school, and engaging with various peer groups on a daily basis, which significantly shaped or influenced their experiences of living with HIV, their emotions, their mental health, and their social relationships in ways that differ notably from adults living with HIV [50]. For instance, the particular settings and situations they were in heightened the challenges of concealing their HIV status from parents and peers. This, in turn, led to increased anxiety and fear regarding the potential for others to uncover information about their health condition or HIV status. The findings highlight the importance of targeted mental health interventions and activities for ALHIV in the Indonesian context, which are currently lacking, to facilitate their emotional regulation and self-acceptance, enhance psychological resilience, and equip them with emotion-focused strategies to cope with mental health challenges [7,40,51]. Evidence from other contexts has shown that mental health interventions are effective in improving the mental health conditions of ALHIV [52]. Similarly, the WHO has suggested that such interventions can strengthen the mental health of these adolescents and lead to improvements in their overall physical and health conditions [53].

This study also highlights a range of family-related factors that contributed to the mental health challenges faced by these adolescents, which have not been reported in previous studies [19,22,23,28]. The dynamics within families of adolescents were a critical factor influencing mental health. Fractured family homes, family conflict, and the subsequent impact on the mental health of adolescents show the importance of familial relationships in shaping emotional responses to the challenges of living with HIV [54]. Similarly, the absence of parental support, due to loss or unacceptance of their HIV status by parents, and non-disclosure of HIV status to parents left them feeling isolated and without the appropriate emotional resources required to cope with the challenges associated with HIV. For some adolescents, the lack of parental acceptance and non-disclosure of HIV status created an unsupportive environment for child-parent open dialogue about HIV. Parents play vital roles in their children’s lives and can be a significant source of emotional support by offering encouragement, expressing affection and admiration, and fostering feelings of safety, which can enable them to deal with various psychological distresses [55,56]. Some studies have reported that parental and familial support positively impacts individuals, particularly children living with HIV, and enhances their engagement and retention in HIV treatment and care [54,57]. Therefore, for some adolescents in this study, the absence of parental support not only exacerbated the mental health challenges they faced but could also hinder their ability to cope effectively with life challenges in general. Some adolescents appeared to intend to conceal their HIV status from their parents and experienced difficulties in relation to that due to their specific condition of living with parents in sharing housing. Given they were still under parental support, interventions that promote open communication between adolescents and parents about HIV status are essential. Such interventions could increase acceptance of the HIV status among adolescents, parents, and family members, potentially leading to the provision of crucial support from parents, wider family members, and others. Mental health interventions aimed at strengthening relationships and communication between parents/caregivers and adolescents have been reported to be effective in bringing about positive changes in the mental health and wellbeing of both ALHIV and their caregivers/parents [58].

This study also highlights stigma and discrimination as significant influencing factors for the mental health challenges faced by ALHIV. The perceived, anticipated, and enacted stigmas that these adolescents were aware of and experiencing highlight the societal attitudes towards HIV that they had to navigate, which were significant drivers of the mental health issues they encountered. Although the association between HIV stigma, discrimination, and mental health issues have been reported in previous studies with ALHIV [19,23,59,60] and adults living with HIV in Indonesia and globally [35,36,61,62], the current findings present the adolescents’ specific family and school environments that shaped and influenced their awareness and experience of HIV stigma, discrimination, and subsequent mental health challenges. Their experiences of stigma and discrimination also hindered their access to education and social integration, seemingly creating a cycle of disadvantage that reinforced their sense of isolation and contributed to a decline in mental health. Additionally, it can be argued that the Indonesian context, where HIV and PLHIV are highly stigmatized within families and communities [35,36], seems to play a pivotal role in influencing their emotions, mental health, social relationships, and behaviors. Our findings indicate the importance of HIV-related health promotion interventions within school settings to enhance the knowledge of students and teachers regarding HIV, to reduce stigma, and to support the acceptance of ALHIV [63,64,65]. Such interventions can also be beneficial in reducing or preventing stigma and promoting inclusion within educational or school settings.

### Limitations and Strengths of the Study

The results of this research must be interpreted with caution due to some limitations. The use of a snowball sampling technique, which began with the dissemination of study information sheets through receptionists and an information board at a healthcare facility providing HIV care services and via a WhatsApp group for adolescents living with HIV, may have introduced a bias by recruiting only ALHIV who shared similar characteristics. This included individuals who had been on HIV treatment and were part of networks of adolescents living with HIV. Consequently, ALHIV who were not part of these networks, and who may have had different experiences and life stories, were not included. This also means that the diversity of the recruited participants is limited. Therefore, the findings reflect the specific experiences of the participants, which may not be applicable to other ALHIV with different characteristics. Despite these limitations, the strength of the study lies in its being the first qualitative investigation to explore mental health challenges and contributing factors among ALHIV in the Indonesian context [11]. As such, the findings provide valuable insights that can inform HIV policies and interventions aimed at addressing the needs and challenges faced by ALHIV in the study setting, as well as in Indonesia and beyond.

## 5. Conclusions

This study presents complex and multifaceted mental health challenges and their contributing factors among ALHIV in Yogyakarta, Indonesia. It demonstrates that their HIV-positive status, intertwined with family-related factors, stigma, and discrimination, plays significant roles in influencing participants’ mental health conditions. Given the Indonesian context where interventions to address HIV impact among ALHIV are lacking, this study’s findings indicate a need for tailored and targeted intervention programs, as previously mentioned, to support mental health, reduce stigma, and promote child-parent open communication on HIV and HIV disclosure in safe ways for ALHIV. Future quantitative studies involving a large number of ALHIV, in order to investigate the complexity of mental health challenges they faced and the broader contributing factors at individual, familial, and societal levels in the context of Indonesia, are recommended, as their results could be generalized to other population groups and could better inform the development of HIV policies and intervention programs to support their mental health, address reduce stigma, and create a safe environment for HIV communication and disclosure.

## Data Availability

The data presented in this study are available on request from the corresponding author. The data are not publicly available due to restrictions set by the human research ethics committee.

## Supplementary Materials

The following supporting information can be downloaded at: https://www.mdpi.com/article/10.3390/ijerph22010083/s1, File S1. Interview Guide.
