# Supplementary material for: Understanding HIV-Related Mental Health Challenges and Contributing Factors Among Indonesian Adolescents Living with HIV
Source: Int J Environ Res Public Health. 2025 Jan 9;22(1):83. doi: 10.3390/ijerph22010083 (PMC11764498; doi:10.3390/ijerph22010083)
Supplement: Supplementary file 1 [file ijerph-22-00083-s001.zip › ijerph-3343508-supplementary.pdf]

TORRENS UNIVERSITY AUSTRALIA

Interview guide: Adolescents living with HIV in Yogyakarta, Indonesia

Date: ______________________________(Date)
Interviewer: ____________________ (Name)
Interviewee: ________(Code);

Is it OK if I record the interview and take notes during the interview?

When I write up the results of this study, I will assign a specific study identification letter and number for you (as well as for each participant I interview) to ensure that the information or comments you provide in this interview remain anonymous. Is this OK with you? You can withdraw your participation or ask me to stop this interview if you do not want to continue or feel uncomfortable during this interview without any consequences.

As you have known through the study information sheet and our initial conversation once you confirmed your willingness to participate, the purpose of this study is to gain your insights into the mental health challenges you face following your HIV diagnosis, factors that contribute to those challenges and your HIV-related needs or expectations.

Demographic information
How old are you? : .... (in years)

Sex :

Age at first HIV diagnosis : .... (in years)

What is the highest education you received? : Not at all/primary/secondary/tertiary

Are any other family members of yours who are living with HIV? :

How do you know about your HIV status?

- Who informed you (your parents/doctors) and how? Please tell me more about this.
- What was the situation when you were informed about your HIV status? Was it at home/hospital? Please explain more about it.
- Who else in your family knows about your HIV status and how do they know?

What went through your mind or how did you feel when you were informed about your HIV status for the first time?

- Were you shocked, sad, angry, afraid, stressed, etc, and why? Please tell me more about it.
- For how long did you experience those kinds of feelings?
- What about now, do you still experience those feelings sometimes or they are completely gone? Why? Please tell me more about it.

torrens.edu.au
1300 575 803
Adelaide Campus
82-98 Wakefield Street
Adelaide SA 5000
Brisbane Campuses
90 Bowen Terrace
Fortitude Valley QLD 4006
123 Gotha Street
Fortitude Valley, QLD 4006
Melbourne Campuses
196 Flinders Street
Melbourne VIC 3000
25 Victoria Street
Fitzroy VIC 3065
Sydney Campuses
1-5 Hickson Road
The Rocks NSW 2000
Level 3, 333 Kent Street
Sydney NSW 200
46-52 Mountain Street
Ultimo NSW 2007
Level 1 and 5/235 Pyrmont Street
Pyrmont NSW 2009
Level 4, 540 George Street
Sydney NSW 2000
Leura Campus
1 Chambers Road
Leura NSW 2780
Torrens University Australia Head Office
Level 24, 680 George Street
Sydney NSW 2000

What about your relationships with your family members (parents and siblings) after they know about your HIV status?

- Are there any differences or changes in their attitudes and behaviours? Tell me more about it.
  - How do they treat your personal belongings, including clothes, eating utensils, etc (stigma/discrimination).
- Do you think your family members' attitudes and behaviours have an influence on your mental health condition? How? Please tell me more about it.
- What about your relationships with your parents, especially when you know that you have mother-to-child transmission?
  - Does it influence you mentally? How? Please tell me more about it.

Please tell me more about your family situation:

- Do all of you get along very well or not? Why? Please tell me more about it.
- What do you think about your family condition: does it have an influence on you psychologically? How? Please tell me more about it.

Do your extended families/relatives know about your HIV status? How do they know?

- How do you feel about their attitudes and behaviours or reactions towards you when they know about your HIV status?
  - Are there any differences? How? Do you know why? Please tell me more about it.
- How do their attitudes and behaviours influence your mental health condition? Please explain more about it.

What do you think about your education and future?

- Do you think the mental health challenges you experience have an influence on your education? How? Why? Please explain more about it.
- How do mental health challenges facing you influence your thoughts about your future? Please explain more about it.
- What are your thoughts about your future?
  - Motivation to have a good job in the future or not? Please tell me about it.

What do you think about the attitudes and behaviours of your neighbours or community members who know about your HIV status?

- Have you noticed any differences?
  - Avoidance, rejection mocking, gossiping, etc…..
- Do you think their attitudes and behaviours have an influence on you mentally?
  - Make you worried, angry, stressed, etc? Tell me more about it
- What about the attitudes and behaviours of your friends at school?
  - Are there any differences that make you feel uncomfortable, worried, etc. Please tell me more about it.
- Do you think your friends' attitudes and behaviours towards you have an influence on your relationships with them? How? Please explain more about it.

In relation to access to HIV care services, have you ever experienced any unpleasant attitudes or behaviours from healthcare professionals (HCPs) in any healthcare facility?

torrens.edu.au
1300 575 803
Adelaide Campus
82-98 Wakefield Street
Adelaide SA 5000
Brisbane Campuses
90 Bowen Terrace
Fortitude Valley QLD 4006
123 Gotha Street
Fortitude Valley, QLD 4006
Melbourne Campuses
196 Flinders Street
Melbourne VIC 3000
25 Victoria Street
Fitzroy VIC 3065
Sydney Campuses
1-5 Hickson Road
The Rocks NSW 2000
Level 3, 333 Kent Street
Sydney NSW 200
46-52 Mountain Street
Ultimo NSW 2007
Level 1 and 5/235 Pyrmont Street
Pyrmont NSW 2009
Level 4, 540 George Street
Sydney NSW 2000
Leura Campus
1 Chambers Road
Leura NSW 2780
Torrens University Australia Head Office
Level 24, 680 George Street
Sydney NSW 2000

- Avoidance, left untreated, unnecessary referrals, …. Tell me more about it.
- Do you think negative attitudes and behaviours from HCPs, if any, have an influence on you psychologically? How? Please explain more about it.

Following your HIV diagnosis, are there any difficulties you experience in your relationships with your friends?

- Difficulties in communicating or socialising with others, feeling ashamed, fear, etc
- Social withdrawal, …. Please tell me more about it.
- What is your experience with others' attitudes and behaviours towards you at your schools or communities? Please explain more.
  - From your teachers
  - From your peers
  - Do you think others' attitudes and behaviours influence your mental health condition? Please explain more.

Is there anything else you wish to add?

Would you be able to suggest other WLHIV who might be willing to participate in this study?

Torrens University Australia Ltd, ABN 99 154 937 005, RTO41343, CRICOS 03389E
